# Characterization of components of resistance to Corn Stunt disease

**DOI:** 10.1371/journal.pone.0234454

**Published:** 2020-10-19

**Authors:** José Darío Oleszczuk, María Inés Catalano, Lucía Dalaisón, Julio Alejandro Di Rienzo, María de la Paz Giménez Pecci, Pablo Carpane

**Affiliations:** 1 Bayer Argentina, Fontezuela, Buenos Aires, Argentina; 2 Centro de Investigaciones en Biología (CEBIO), Pergamino, Buenos Aires, Argentina; 3 Cátedra de Estadística y Biometría, Facultad de Ciencias Agropecuarias, Universidad Nacional de Córdoba, Córdoba, Argentina; 4 Instituto Nacional de Tecnología Agropecuaria (INTA), Instituto de Patología Vegetal (IPAVE), Córdoba, Argentina; Osmania University, INDIA

## Abstract

Corn Stunt is an important disease in the Americas due to it high prevalence and the yield reductions that can cause when present. However, changes in the presence of this disease across years hampers the effective identification of resistant genotypes to this disease. To avoid the limitations of phenotypic selection under natural pressure, this research aimed to devise an effective strategy to screen disease-resistant genotypes in the absence of high and constant natural pressures. To do so, we investigated the presence of antixenosis and antibiosis as components of resistance to the vector *Dalbulus maidis* as well as resistance to the pathogen *Spiroplasma kunkelii* under artificial inoculation conditions in four maize hybrids. The hybrids shown differences in their levels of resistance and target organisms, either the insect vector or the pathogen. Antixenosis and antibiosis to *D*. *maidis* were observed in DK72-10. Resistance to *S*. *kunkelii* by DK79-10 was seen as a delayed onset of symptoms, and DKB390 showed antixenosis to *D*. *maidis* and resistance to *S*. *kunkelii*. An association between symptom severity and yield reduction was found, but not between accumulation of pathogen *S*. *kunkelii* and symptom severity nor yield. In conclusion, the proposed methodology was efficacious and can aid in the screening of resistant genotypes in breeding programs to reduce the impact of Corn Stunt disease, ensuring that hybrids with good resistance level will be planted by farmers whenever disease occurs.

## Introduction

Corn Stunt is one of the most significant diseases affecting maize crop in the Americas, because of its high prevalence and its potential to cause yield losses in endemic areas [1–3]. Following its initial detection [4, 5], its prevalence has increased in the Americas [2, 6–8]. Corn Stunt disease was first confirmed in the subtropical region of Argentina during the 1990/91 crop season [9]. High disease prevalence was later reported in this region in 1996/97, 2001/02, 2006/07 and 2010/11 crop seasons [7, 8, 10], and isolated symptomatic plants may be found occasionally in temperate areas of Argentina [8, 11–13].

The mollicute *Spiroplasma kunkelii* Whitcomb, known as Corn Stunt Spiroplasma (CSS), is the pathogen most commonly associated with Corn Stunt disease in Argentina. This mollicute is transmitted by leafhoppers, being *Dalbulus maidis* (DeLong) the only known vector species found in South America [11, 14–16], although *Exitianus obscurinervis* Stal (Hemiptera: Cicadellidae) was proven to be a vector species in experimental conditions [17]. The epidemiological significance of *D*. *maidis* lies in its high prevalence [18], high transmission efficiency of the pathogen *S*. *kunkelii*, with no reduced longevity [18, 19], and the persistent-propagative transmission mode, so insects acquiring the pathogen remain inoculative throughout their lifespan [20]. In addition to *S*. *kunkelii*, *D*. *maidis* is also vector of two other pathogens: Maize Bushy Stunt Phytoplasma (MBSP) [20] and Maize Rayado Fino virus (MRFV) [21], which can be also present in field.

Symptoms of Corn Stunt disease appear three to five weeks after inoculation of *S*. *kunkelii* and become more severe with time and to the newest parts of the plants as they develop. Mild symptoms include typically leaves with chlorotic stripes that appear near the base and extend towards the leaf tips [20, 22]. Alternatively, leaves may show reddening [3] or deformations (“cuts”) in the margins. Severe symptoms appear as shortened internodes, which gives its name to stunting, and in some cases ear proliferation or even lack of reproductive structures of the plant [20, 23]. Reduced yield resulting from Corn Stunt disease is directly related to symptom severity and the accumulation of the pathogen *S*. *kunkelii* [24], which are both highest if *S*. *kunkelii* is inoculated at early growth stages [25, 26]. In this situation, reduced yield may be high, ranging from 12 to 100% [25–28].

One of the most convenient alternatives to reduce yield loss resulting from diseases is the use of resistant crops [29]. The resistance of maize genotypes to *S*. *kunkelii* might remain stable over time and across regions due to the low genomic variation of this pathogen [30]. Field-resistant genotypes have in fact been obtained in areas of high pressure [31–36]. However, the rare incidence of Corn Stunt disease in Argentina hinders the effective identification of resistant genotypes to this disease. The accurate detection of resistant genotypes may play a key role in the management of Corn Stunt disease in areas where the planting of temperate genotypes (obtained in areas where the prevalence of Corn Stunt is low or null) has increased over the past years ahead of tropical genotypes, which should *a priori* be more resistant to Corn Stunt for being obtained in a disease endemic area.

In lack of high and constant natural pressures, the analysis of resistance mechanisms to Corn Stunt may be effective to select resistant genotypes [37, 38], with the possibility to identify and combine these resistance mechanisms. In similar pathosystems, plant resistance may be targeted to the insect vector or to the pathogen [37]. Plant resistance to insects may be composed of two elements: antixenosis and/or antibiosis [39, 40]. In antixenosis, the plant is a non-preferred host for insects, decreasing the contact time between them, and hence the efficiency of pathogen transmission. Antibiosis occurs after the plant has been colonized by insects and reduces feeding, development, survival and reproduction. This would lower the rate of population increase of insect vectors, lessening the magnitude of secondary disease spread in the field. Conversely, plant resistance could be aimed to the pathogen, since maize genotypes resistant to Corn Stunt show milder symptoms and lower yield reduction [33, 41, 42].

The goal of this research was to identify and characterize resistance mechanisms to Corn Stunt disease in maize hybrids from temperate and tropical regions of Argentina, aiming to generate a screening methodology to be used in the absence of high Corn Stunt pressures.

## Materials and methods

The tests implemented investigated the existence of different resistance mechanisms to Corn Stunt disease: antixenosis and antibiosis to vector *D*. *maidis* [40] and resistance to pathogen *S*. *kunkelii* [29].

### Biological materials

A colony of healthy *D*. *maidis* was initiated from insects collected in the province of Tucumán (located in the tropical area of Argentina) and was maintained on plants of sweet corn variety Maizón at the IPAVE-CIAP (Plant Pathology Research Institute—Center of Agricultural Research (IPAVE-CIAP) at INTA (National Institute of Agricultural Technology) Córdoba, Argentina and at CEBIO (BioResearch Center) at UNNOBA-CICBA (National University of the North West of the Province of Buenos Aires—Scientific Research Commission of the Province of Buenos Aires), Pergamino, Buenos Aires, Argentina. The colonies were kept in aluminum-framed cages with a “*voile*” type nylon mesh, placed in a growth chamber at a temperature of 25°C, with a photoperiod of 16:8 (light: darkness) hours [20].

Four maize hybrids were used for this study: DK670 and DK72-10 from the temperate region of Argentina, and DK79-10 and DKB390 from the tropical region of Argentina. The seeds were obtained directly from Bayer’s seed processing facilities before they were treated, and for this reason they had no insecticides nor fungicides.

### Preference test (antixenosis to the vector *Dalbulus maidis*)

#### Test conditions

The test was performed under controlled conditions at CEBIO at a mean temperature of 20–30°C using seeds planted in pots (1 seed per pot).

Plants with two fully expanded leaves were used. A plant from each hybrid was transferred into a glass cage. Pots were placed horizontally on the cage floor, so plants could be mounted in such a way that they exposed only a single leaf with its abaxial side upwards. Six two-week-old adult *D*. *maidis* mated females were later introduced into the cage.

Preference was determined by recording the number of insects settled on each hybrid or not settled on any hybrid at 1, 6, 24 and 48 hours (timepoints) after their release. Leaves were dissected at the end of the test. The number of eggs laid was counted under a binocular microscope.

#### Experimental design and statistical analysis

The experiment was replicated 50 times, with each cage of four hybrids and six females serving as a replication. The arrangement of hybrids was randomized across replications. For the statistical analysis, a multinomial model was adjusted modeling hybrid and timepoints effects. The model was adjusted using the nnet package [43] of R language [44]. The response variable was the proportion of insects settled on each hybrid or on the cage. The significance of the differences in intercepts and slopes in the interaction was analyzed with contrasts using this same module. A generalized linear mixed model was used for oviposition to model negative binomial variables, with hybrid as fixed effect and replication as random effect.

### Survival test (antibiosis to the vector *Dalbulus maidis*)

#### Test conditions

The test was performed under controlled conditions in IPAVE-CIAP at a temperature of 20-30°C using seeds planted in pots (1 seed per pot).

The test was conducted in plants with two fully expanded leaves. Each replication consisted of four polyethylene cages each containing a plant from one of the hybrids. Five two-week-old adult *D*. *maidis* mated females were then released. The number of surviving insects was counted weekly for four weeks (timepoints), and plants were replaced to ensure the presence of fresh plants.

#### Experimental design and statistical analysis

The experiment was replicated 30 times, being a replication each group of four polyethylene cages containing each cage one hybrid and five insects. The distribution of cages was randomized across replication. For the statistical analysis, a generalized linear mixed model was used, being hybrid, timepoint and interaction fixed effects. The response variable was the probability of survival, with binomial distribution and a logit linkage function.

### Symptom progression test (resistance to the pathogen *Spiroplasma kunkelii*)

To prevent reduced inoculation efficiency of pathogen *S*. *kunkelii* due to the potential presence of antixenosis and antibiosis to vector *D*. *maidis* by the hybrids tested, the artificial infestation took place using a high pressure of inoculative insects in no-choice conditions [19, 37]. This was considered enough to ensure that *S*. *kunkelii* was indeed inoculated to all plants as in other pathosystems [45].

#### Test conditions

A population of inoculative *D*. *maidis* was initiated by collecting symptomatic plants in Las Breñas, Province of Chaco, which were taken to IPAVE-CIAP. The acquisition of *S*. *kunkelii* from these plants and its subsequent maintenance was conducted according to Nault [20] and adapted by Carpane [38], in terms of acquisition access (7 days), incubation (21 days) and inoculation access (7 days) periods. Before the test, inoculative *D*. *maidis* were placed in glass test tubes (height: 15 cm; diameter: 2 cm) in groups of six adult insects and were transferred into the field where inoculation took place. Insects were not sexed, because transmission efficiency is similar in both genders [19].

Inoculation was performed in a field close to Monte Cristo, Province of Córdoba, at 30 km from IPAVE-CIAP. The presence of Corn Stunt disease in this area is typically low or null, thus minimizing the risk of natural infections from *D*. *maidis* interfering with forced inoculation. Plots of nine 500-meter-long rows were planted of each hybrid, with a row spacing of 52 cm and a density of 3.5 seeds per linear meter. At the four-leaf stage, five homogeneous blocks were labeled, and five plants of similar size were selected in each of them and individually covered with a “*voile*” type nylon cage. Insects were released into each cage (a tube containing six insects per plant) for an inoculation access period (IAP) of 48 hours, long enough to obtain maximum inoculation efficiency of *S*. *kunkelii* [19]. As negative control, five plants of each hybrid were exposed to insects from the healthy colony (six adults per plant). Following the IAP, insects were controlled with insecticides and cages were removed. Insecticides were periodically sprayed later to prevent natural inoculation of *S*. *kunkelii* by eventual populations of *D*. *maidis* or by insects hatching from eggs laid in inoculated plants.

#### Progression of disease incidence and severity

Symptom severity was assessed at 20, 45, 65 and 85 days after inoculation (DAI) timepoints. A 0–4 grade scale based on Carpane et al. [23] was used for the assessment, where 0 = no symptoms, 1 = leaves with red margins (red leaves), 2 = leaves with chlorotic stripes, 3 = leaves with mild stunting (height 15–30% of non-inoculated plants), 4 = leaves with severe stunting, with a height lower than 30% of non-inoculated plants.

#### Detection of the pathogen *S*. *kunkelii*

In the last timepoint of symptom assessment (85 DAI), tissue samples from the ear leaf and the penultimate leaf from the tassel were collected to diagnose the presence of *S*. *kunkelii* at IPAVE-CIAP. A sample of 0.5 g leaf was cut and macerated in 5 mL of Phosphate-Buffered Saline with Tween 20 (PBST), according to Shibata et al. [45]. The accumulation of *S*. *kunkelii* was estimated using a double antibody sandwich enzyme-linked immunosorbent assay (DAS-ELISA) conjugated with alkaline phosphatase. *S*. *kunkelii* accumulation was expressed as relative absorbance (RA), this being the ratio between absolute absorbance at 405 nm of each leaf sample and a threshold of absorbance (mean + 3 standard deviations of absolute absorbance) obtained from six healthy plants of each hybrid [46]. Plants were considered positive (with presence of the pathogen *S*. *kunkelii*) if RA was higher to 1 in any of the leaves tested.

#### Determination of yield

When grain moisture of DK72-10 (intermediate relative maturity of the hybrids tested) reached 16%, ears from each plant were harvested individually to determine yield (g/plant), which was expressed as qq/Ha, with an estimated planting density of 70,000 plants/Ha and a grain moisture of 14.5%. In turn, to neutralize the effect of yield potential *per se* of the hybrids tested, relative yield was calculated as the ratio between the yield of each plant and the average yield of ten non-inoculated plants from the same hybrid (all these plants were diagnosed negative for *S*. *kunkelii*) and expressed as percentage.

#### Experimental design and statistical analysis

The statistical analysis of symptom progression was performed using a generalized mixed linear model with hybrid, timepoints (DAIs) and interaction as fixed effects. The response variables were binary (logit linkage): the incidence of plants with symptoms and plants with severe symptoms (grades of 3 or 4 in the scale mentioned above). The number of plants with a positive diagnose in DAS-ELISA was analyzed as the symptom progression considering only hybrid as fixed effect since samples were taken only once for diagnosis, and the incidence of plants with positive diagnosis in the ear leaf and the penultimate leaf as response variables. Accumulated severity (AS) of each plant was calculated by summing the severity grades across timepoints (DAIs). AS was analyzed in the same way as the number of plants with positive diagnose in DAS-ELISA. The relationship between AS and yield with RA was analyzed with Spearman’s rank correlation, including only diseased plants (either showing symptoms or positive diagnosis in DAS-ELISA).

Yield was analyzed using a mixed linear model, with hybrid as fixed effect and block as random effect. Assumptions were validated through graphical analyses (residuals vs. predicted, normal QQ plot). Response variables were yield (qq/Ha) and relative yield (%). The relation between yield and AS was analyzed with mixed linear models, with hybrid, AS and their interaction as fixed effects. Linear models and generalized mixed models were adjusted using nlme [47] and lme4 [48] packages of the R language [44] through the statistical software interface InfoStat [49]. Predicted values were compared using DGC test [50], with a significance level of 5% for all cases.

## Results

### Preference (antixenosis to the vector *Dalbulus maidis*)

**Fig 1:**
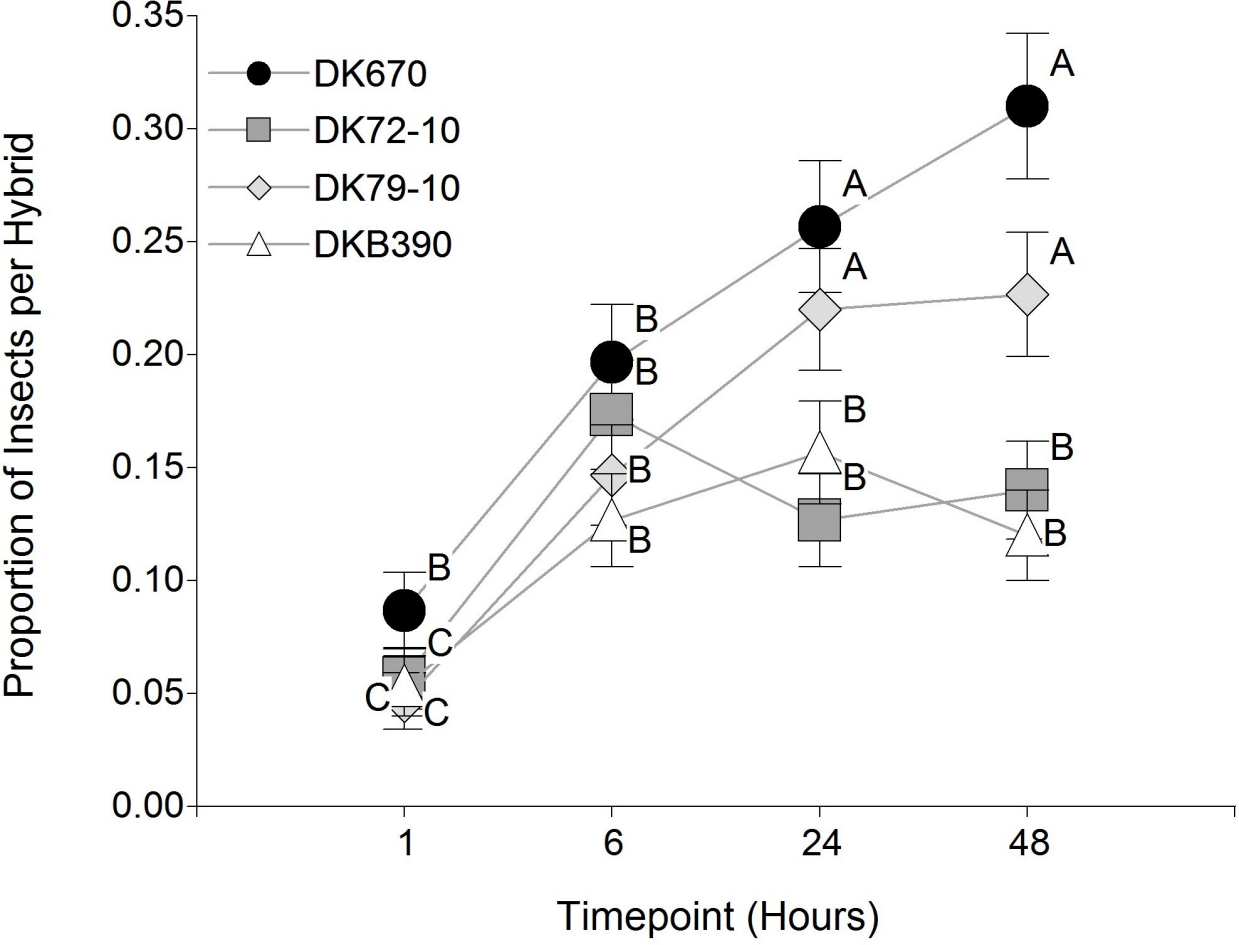
Proportion of *Dalbulus maidis* individuals settled on different hybrids over time. Values sharing the same letter are not statistically different for a 5% significance level. Values with the same letter are not significantly different according to contrasts in the multinomial test (α = 0.05). Bars indicate standard error of the mean.

In the settling preference of adult *D*. *maidis* (Fig 1), a significant effect of hybrid and timepoint factors was observed, as well as their interaction (p<0.0001 in all cases). The hybrid effect was due to a higher number of insects settled on DK670 and DK79-10 than on DK72-10 and DKB390. The timepoint effect resulted from the increase in the number of insects settled on hybrids over time, as most insects settled on the cage rather than on hybrids at the first timepoints (mainly in the first hour of the test). The interaction between hybrid and timepoint (p = 0.0213) was due to insects settling more rapidly over time in DK670 and DK79-10 than in DK72-10 and DKB390.

**Fig 2:**
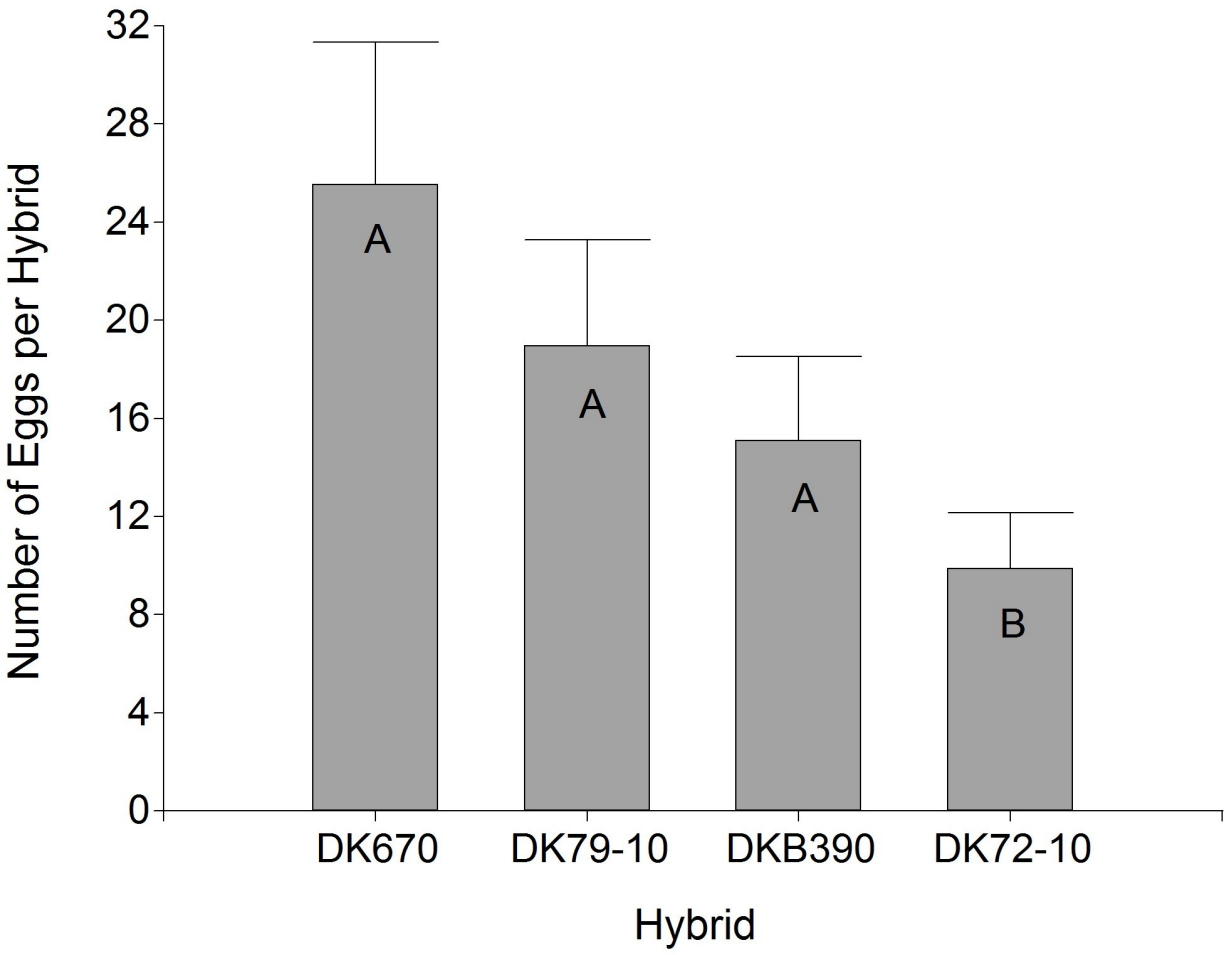
Number of eggs oviposited by *Dalbulus maidis* females in four maize hybrids. Values with the same letter are not significantly different according to contrasts in the mixed model test (α = 0.05). Bars indicate standard error of the mean.

The number of eggs laid by *D*. *maidis* females on each hybrid (Fig 2) showed a significant effect of the hybrid factor (p<0.0001), with a similar arrangement to Fig 1, except that only DK72-10 had significantly fewer eggs than the other hybrids.

### Survival (antibiosis to the vector *Dalbulus maidis*)

**Fig 3:**
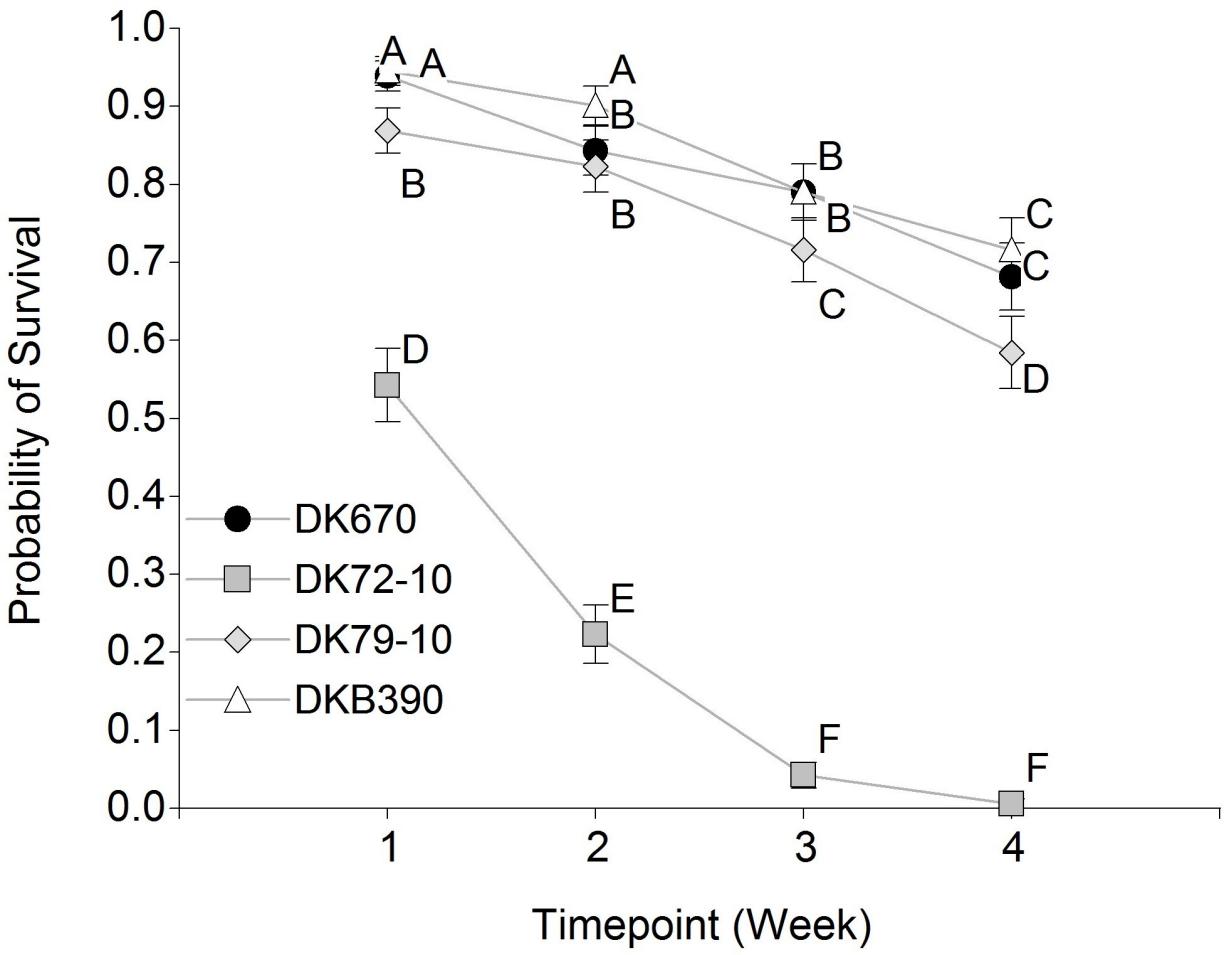
Probability of survival of *Dalbulus maidis* adults over time in four maize hybrids. Values sharing the same letter are not statistically different for a 5% significance level. Values with the same letter are not significantly different according to contrasts in the mixed model test (α = 0.05). Bars indicate standard error of the mean.

For the probability of survival of *D*. *maidis* adults over time (Fig 3), a significant effect was seen for the hybrid and timepoint factors, as well as their interaction (p<0.0001 in all cases). The hybrid effect was due to the less survival in DK72-10 compared to other hybrids, and the timepoint effect resulted from the reduced survival over time in all hybrids. In turn, the interaction between hybrid and timepoint is explained by the faster decrease in survival in DK72-10 than in the other hybrids.

### Symptom severity (resistance to pathogen *Spiroplasma kunkelii*)

#### Progression of disease incidence and severity

**Fig 4:**
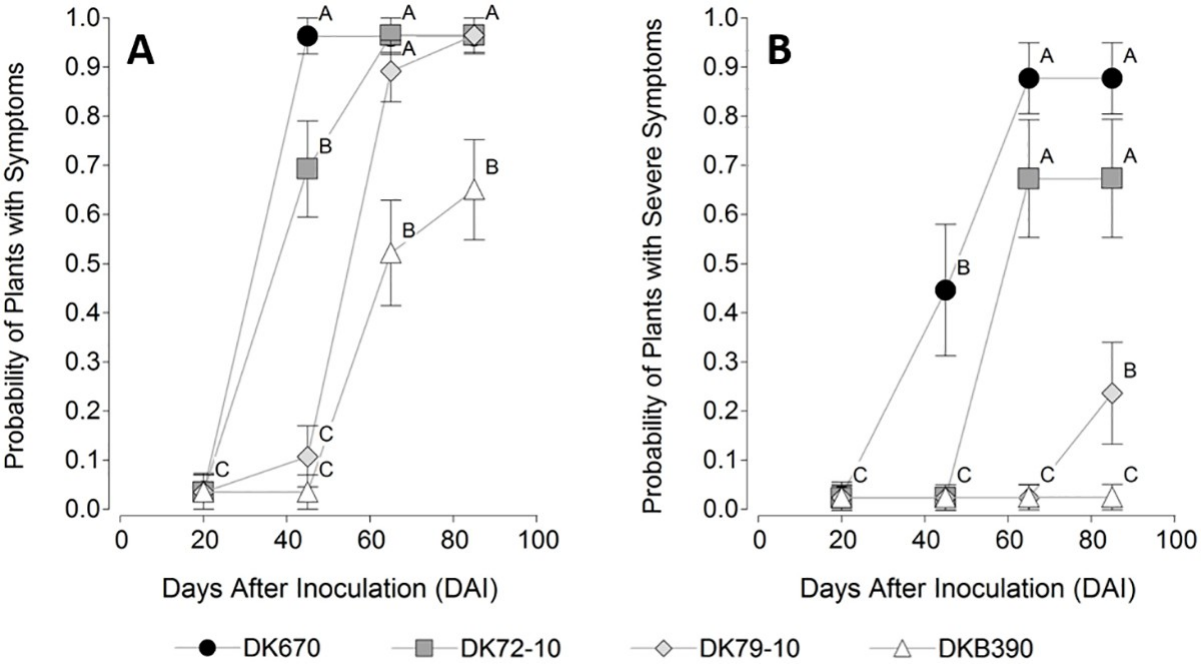
Incidence (probability) of symptomatic plants inoculated with *Spiroplasma kunkelii* to four maize hybrids. (A) plants with mild + severe symptoms, (B) plants with severe symptoms. Values sharing the same letter (within each panel) are not statistically different for a 5% significance level. Values with the same letter are not significantly different according to contrasts in the mixed model test (α = 0.05). Bars indicate standard error of the mean.

The incidence of plants with symptoms (Fig 4A) showed a significant effect for hybrid (p<0.0001) and timepoint (p<0.0001) factors, as well as their interaction (p = 0.0468). The hybrid effect was due to the incidence following the sequence DK670 = DK72-10 > DK79-10 > DKB390, the timepoint effect to the increase of incidence over time, and the interaction to a difference in the rate of increase of the proportion of plants with symptoms, following the order DK670 > DK72-10 > DK79-10 > DKB390. For instance, 96% of plants of DK670 and 68% of plants in DK72-10 showed symptoms at 45 DAI, while only 12% of plants of DK79-10 showed symptoms, and no symptomatic plants were found in DKB390 at this timepoint. Most plants developed symptoms after this period in these last two hybrids, with a higher rate in DK79-10 than in DKB390, which resulted in a higher final (at 85 DAI timepoint) incidence in the former hybrid than in the latter hybrid.

The incidence of plants with severe symptoms (Fig 4B) showed a significant effect of the hybrid (p<0.0001) and timepoint (p<0.0001) factors, as well as their interaction (p = 0.0483). The effect of the individual factors and their interaction was like that described for incidence of plants with mild + severe symptoms (Fig 4A). At the end of the study, no DKB390 plants had severe symptoms.

**Fig 5:**
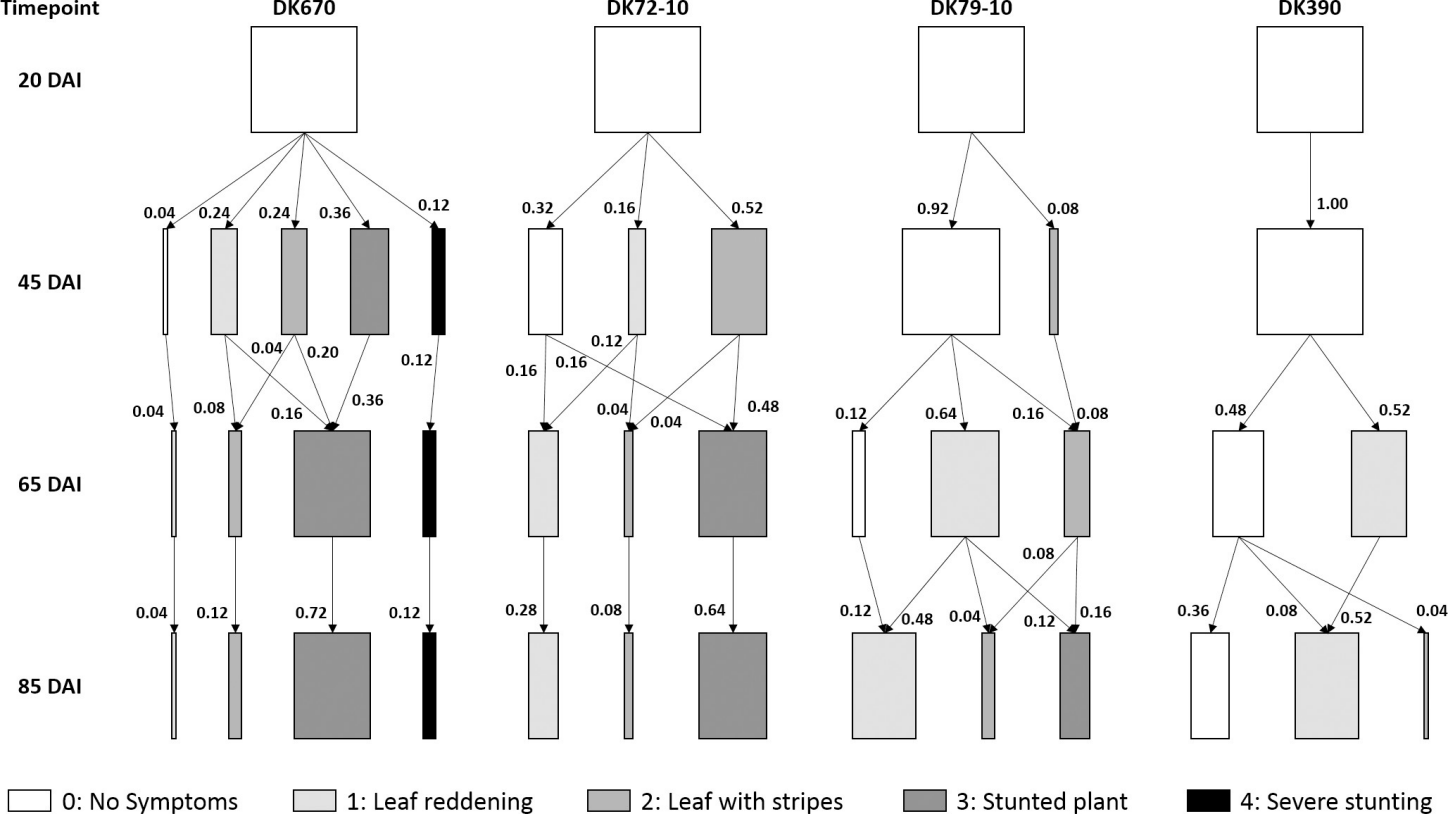
Kinematic diagram of the sequence of Corn Stunt symptoms. Four maize hybrids were inoculated at the four-leaf stage.

Fig 5 shows the sequence of symptom progression over time on a plant by plant basis. The hybrids tested had a similar sequence of symptoms, but with differences between them in the time of first detection and the following rate of progression. Symptoms started mostly as leaves with red margins or chlorotic stripes, followed by stunting. In some cases, symptoms were first seen as leaves with red margins followed by chlorotic stripes, although this sequence was less common. Finally, no remission of symptoms was seen in any case, i.e. plants showing symptoms at a certain timepoint kept displaying symptoms later, either similar or more severe.

#### Detection of the pathogen *S*. *kunkelii*

The comparison between the presence of Corn Stunt measured as symptoms and diagnosis using DAS-ELISA was performed on the penultimate (upper) leaf as it was somewhat more related with symptom severity than the ear leaf, mainly in plants that showed only mild symptoms after 65 DAI (Table 1). Four plants out of 100 tested negatives using the ear leaf and positive with the upper leaf (Table 1). Relative absorbances were almost 1 in the ear leaf in two plants (plant #9 in DK79-10 and #4 in DK390), and so the difference in diagnosis comparing the two leaves could be related to experimental error. However, there was a large difference in relative absorbance between both leaves for the other two plants, likely related to lack of detection of *S*. *kunkelii* in the ear leaf of these plants, which had displayed symptoms at the end of the test.

**Table 1:** Comparison of *Spiroplasma kunkelii* diagnosis based on symptom detection and DAS-ELISA.

Hybrid	Plant ^#^	Relative Absorbance (RA)^#^		Symptom Severity (at DAI)			
		Ear leaf	Upper leaf	20	45	65	85
DK79-10	9	0.903	1.561	0	0	0	1
	11	0.375	3.005	0	0	1	3
DKB390	4	0.935	1.114	0	0	0	0
	19	0.126	1.703	0	0	0	1

Plants shown here differed in the result of the diagnosis for based on the ear leaf versus the upper leaf in DAS-ELISA tests, together with progression of symptom severity in these plants.

# RA values below 1 indicate negative diagnosis (*S*. *kunkelii* not found), and above 1 positive diagnosis (presence of *S*. *kunkelii*).

All plants of DK670 and DK72-10 had symptoms and positive diagnosis for *S*. *kunkelii* (Table 2). In DK79-10, 92% of plants had symptoms and positive diagnosis, while 8% of plants with symptoms were diagnosed negative. These plants showed the first symptoms at 85 DAI as reddening of leaf margins (the mildest symptoms). All plants with symptoms in DKB390 (64%) tested positive. In addition, 20% of plants had positive diagnosis but no visible symptoms. Relative absorbances (RA) of these plants were low in the penultimate leaf (average 1.5) and lower to 1 in the ear leaf (that would have tested negative if only this leaf had been used for diagnosis). The remaining 16% of plants of this hybrid had no symptoms and tested negative for *S*. *kunkelii*.

**Table 2:** Summary of *Spiroplasma kunkelii* diagnosis.

Hybrid	Symptoms^#^	Diagnosis	% Plants
DK670	Positive	Positive	100
DK72-10	Positive	Positive	100
DK79-10	Positive	Positive	92
		Negative	8
DKB390	Positive	Positive	64
	Negative	Positive	20
		Negative	16

Proportion of plants with Corn Stunt symptoms and diagnosis for *Spiroplasma kunkelii* through DAS-ELISA after forced inoculation at the four-leaf stage in four maize hybrids.

# Symptoms evaluated at 85 DAI.

The relation between AS and Yield with RA (Table 3) showed a low correlation (lack of association) for all hybrids except DKB390 for AS. A positive and significant correlation of AS with RA was observed in this hybrid, thereby showing that more severe symptoms were associated with a greater accumulation of *S*. *kunkelii*.

**Table 3:** Correlation of yield and symptom severity with pathogen accumulation.

Hybrid	n	AS and RA		Yield and RA	
		r Spearman	p value#	r Spearman	p value#
DK670	24	0.0617	0.7747	0.0718	0.7389
DK72-10	25	-0.0739	0.7254	-0.2085	0.3071
DK79-10	25	-0.0090	0.9659	-0.256	0.2156
DKB390	21	0.5070	0.0190	-0.3857	0.0845

Spearman’s rank correlation between Corn Stunt accumulated severity (AS) and Yield with the accumulation of *Spiroplasma kunkelii* estimated as relative absorbance (RA) using DAS-ELISA in four maize hybrids.

# P values below 0.05 were considered statistically significant in Spearman’s rank correlation test.

### Determination of yield

The yield of inoculated plants (Table 4) showed a significant effect for the hybrid factor (p<0.0001) and was directly correlated to incidence and severity of symptoms (Figs 4 and 5) in the sequence DKB390 > DK79-10 > DK72-10 > DK670. The hybrid effect was also significant in terms of relative yield (p<0.0001), with a sequence DKB390 > DK79-10 > DK72-10 = DK670. The sequence of hybrids in both yield and relative yield is inversely related to accumulated severity; i.e. the hybrids with the highest yield had the lowest accumulated severity (AS), as shown in Table 4.

**Table 4:** Summary of yield and symptom severity.

Hybrid	Yield (qq/Ha)		RY (%)^#^		AS (Rate)*	
DKB390	127.1	A	82.8	A	1.3	D
DK79-10	92.4	B	50.2	B	3.0	C
DK72-10	67.4	C	40.2	C	5.9	B
DK670	46,2	D	37.1	C	8.0	A

Yield (qq/Ha), relative yield (RY, %) and accumulated severity (AS, rate) of four maize hybrids inoculated with *Spiroplasma kunkelii*.

# RY. Relative yield to non-inoculated plants.

* AS. Sum of rates in four timepoints (maximum value of 16, as if severity rating was of 4 in every timepoint).

Values sharing the same letter (within each column) are not statistically different for a 5% significance level. Values with the same letter are not significantly different according to contrasts in the mixed model test (α = 0.05).

**Fig 6:**
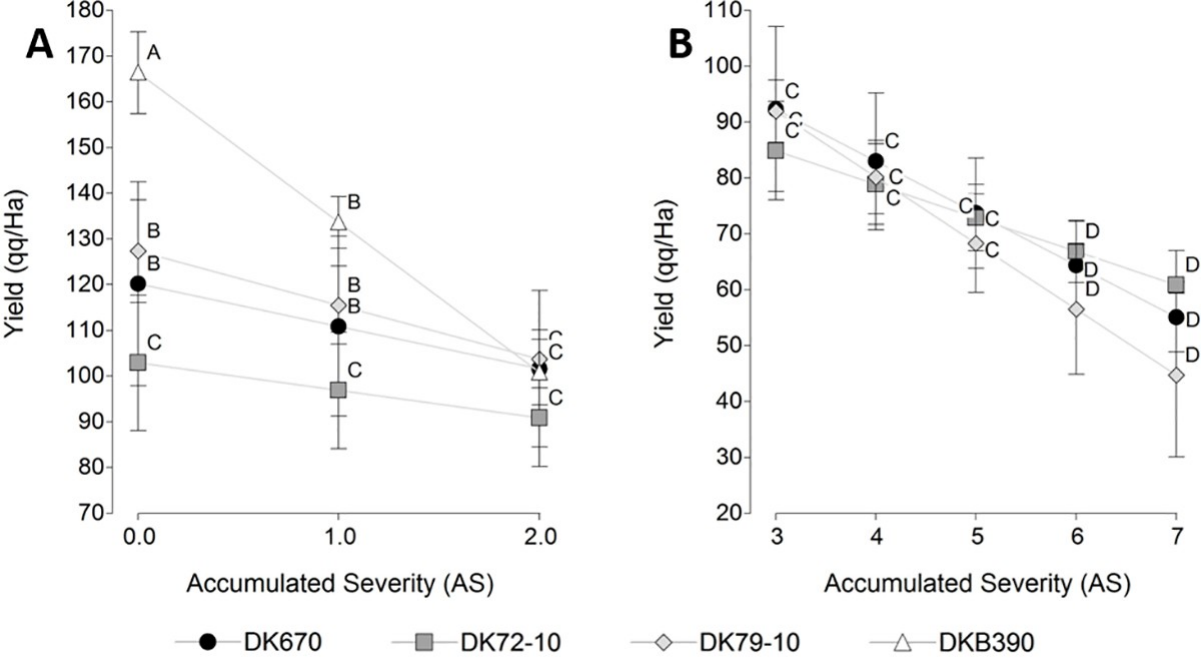
Relation between yield (qq/Ha) and accumulated severity (AS) of corn stunt disease symptoms. (A) Section with AS 0–2 in four maize hybrids. (B) Section with AS 3–7 excluding DKB390, because AS values were lower than 2. Values with the same letter within each panel are not significantly different according to contrasts in the mixed model test (α = 0.05). Bars indicate standard error of the mean.

The effect of symptom severity on yield (Fig 6) was compared in two sections considering that AS was below 2 in DKB390. Fig 6A analyzed the four hybrids in an AS from 0 to 2, and the Fig 6B compared the three remaining hybrids (excluding DKB390) in an AS from 3 to 7. In Fig 6A, there was a significant effect for the hybrid (p = 0.0013) and AS (<0.0001) factors, as well as their interaction (p = 0.0008). The hybrid effect was due to the higher yield of DKB390, the AS effect to decreased yield resulting from the increase in AS, and the interaction to DKB390 having a higher yield than the others in AS of 0–1, without significant differences in an AS of 2. The coefficient of AS factor was -9.8, so was lowered by 9.8 qq/Ha for each unit of increase in AS. In Fig 6B, only AS had a significant effect (p<0.0001), but not the hybrid (p = 0.4345) nor the interaction between both factors (p = 0.3428). The coefficient of the AS factor was -9.3 for this case, indicating that for each unit of increase in AS yield was reduced by 9.3 qq/Ha.

## Discussion

Findings from this research prove that hybrids differ in both their level of resistance to Corn Stunt as well as the target organisms (either the insect vector or the pathogen) and the mechanisms of such resistance. Differences among hybrids were found in the level of antixenosis to *D*. *maidis*, resulting in decreased settling preference of *D*. *maidis* females, like findings in rice with *Nephotettix virescens* [51, 52] and *Sogatella furcifera* [53, 54], and in oats with *Delphacodes kuscheli* [55], where settling preference was also seen as early as six hours from exposure to plants. Our results suggest that maize hybrids DK72-10 and DKB390 negatively interfered with host acceptance by vector *D*. *maidis*, and hence may decrease the inoculation efficiency of the pathogen *S*. *kunkelii* to them, as such efficiency increases with the interval of insect-plant interaction [19]. Settling preference tests using inoculative insects may confirm or rule out this hypothesis, which may be a future line of research.

Only DK72-10 showed antibiosis by reducing survival of *D*. *maidis* relative to other hybrids. An effect of antibiosis was also found in rice against *N*. *virescens* [51, 52], *N*. *cincticeps* [56], *Nilaparvata lugens* [55, 57] and *S*. *furcifera* [53, 58]. However, the antibiosis against *D*. *maidis* seen in this study was lower in absolute terms compared to other cases. For example, survival of *N*. *virescens* adults [51, 52] and *S*. *furcifera* [58] was 20% in five days, while 58% of insects exposed to DK72-10 (the only one having antibiosis) survived one week. Hence, given that survival surpassed the 48-hour period in which maximum inoculation efficiency is achieved [19], antibiosis of DK72-10 would not decrease inoculation rate of *S*. *kunkelii*, although this antibiosis effect of maize against *D*. *maidis* may be present in other genotypes not tested here.

Regarding symptom progression in forced inoculation tests, we analyzed first inoculation efficiency among hybrids and how it was best diagnosed. In this sense, there was a highly close relation between diagnosis by symptom determination and DAS-ELISA, with a higher correlation when using the closest leaf to the tassel for the latter test. This is consistent with previous reports [59, 60] who stressed that the probability of detection of *S*. *kunkelii* was higher in the apex of the plant, which may be explained by the fact that *S*. *kunkelii* moves with photosynthates to the actively growing apical regions of the plant, or that these parts promote the multiplication of *S*. *kunkelii*. In DK670 and DK72-10, all inoculated plants had visible symptoms and tested positive to *S*. *kunkelii*, but some DK79-10 plants that showed their first symptoms in the last timepoint were tested negatives. This may be due to the irregular distribution of *S*. *kunkelii* within the various plant organs and at low concentrations while displaying the first symptoms, resulting in a negative diagnosis [59].

Most plants in DKB390 (64%) showed symptoms and tested positive for *S*. *kunkelii*, but 20% of plants showed no symptoms despite a positive diagnosis, and 16% of plants had no symptoms and a tested negative. On one hand, the presence of plants without symptoms but with positive diagnosis agrees with reports [59], in which *S*. *kunkelii* is detected 10–30 days before symptom onset. Here, these plants might have shown symptoms in a later timepoint, although the test was completed when all hybrids were at physiological maturity. On the other hand, the presence of plants without symptoms and negative diagnosis may be due to: a) a failure in methodology, which seems unlikely because inoculation efficiency was 100% in other hybrids; b) antibiosis and/or antixenosis to *D*. *maidis*, lowering the length of insect-plant interaction and hence reducing inoculation efficiency of *S*. *kunkelii*, although DK72-10 had similar antixenosis and more antibiosis than DKB390 and all of its plants were inoculated, and in similar pathosystems [45, 61] these effects were neutralized using a load of inoculative insects similar to that used in this study; c) resistance of this hybrid to *S*. *kunkelii*, which may reduce movement or replication of this pathogen in the plant following inoculation, leading to *S*. *kunkelii* levels below the limit of detection of the method used [59]. In support of this latter motive, similar results were found for *S*. *kunkelii* in maize [33, 60, 62] and for RTBV and RTSV in rice [37, 63], whom suggest that the target of resistance of some plant genotypes is the pathogen itself rather than the insect vector.

Hybrids differed in symptom progression, including initial detection, subsequent progress and final severity. Symptoms appeared earlier and progressed faster in susceptible vs. resistant hybrids as reported before [26, 62]. Based on these characters, the order of resistance to *S*. *kunkelii* was defined as DKB390 > DK79-10 > DK72–10 > DK670. Hybrids from temperate areas, DK670 and DK72-10, were highly susceptible to *S*. *kunkelii*, as symptoms developed early (although later in DK72-10) and progressed rapidly reaching a high proportion of plants with severe damage and low yield. Symptoms in DK79-10 appeared later and progressed slowly, leading to a lower rate of plants with severe symptoms and intermediate yield. Finally, DKB390 was the most resistant hybrid to *S*. *kunkelii*, as some plants had no symptoms and no pathogen was found in them, others had no symptoms despite the presence of *S*. *kunkelii*, and others developed late-onset mild symptoms, resulting in the highest yield. These results confirm that higher yield reductions occur when symptoms appear early and progress rapidly as discussed before [25, 26, 33, 60], leading to two major concepts related to the control of this disease: a) the need to control Corn Stunt at early growth stages, way before symptom appearance, b) to identify resistant genotypes, rating symptom progression may be more useful than a single assessment at a specific timepoint. In this sense, an aspect to consider is that the lack of a complete correlation between symptom presence and diagnosis suggests that both are necessary to properly characterize genotypes resistant to Corn Stunt disease.

Yield loss caused by *S*. *kunkelii* ranging from 20% to 63% with final incidence rates between 63 and 100% are within the range previously reported [24–27, 33, 61]. These authors also discussed that genotypes with similar levels of incidence and severity of symptoms can achieve different yields [26, 33, 60]. This has been described as host tolerance, or the ability to obtain yield despite the damage caused by Corn Stunt. However, this response was not seen in this study, as yield reductions were directly related to the incidence and severity of symptoms.

No consistent relation was found between the accumulation of *S*. *kunkelii* and symptom severity, both intra- and inter hybrids. This contradicts other results [24, 40, 59] reporting a higher accumulation in plants with severe symptoms but is consistent with findings in rice [45], where no differences were observed in the accumulation of viruses causing Rice Tungro Disease among varieties differing in resistance or among plants with varying severity. The absence of a correlation in this work may be due to the method used (DAS-ELISA), which was performed here without a dilution curve to estimate the concentration of *S*. *kunkelii*. In turn, new methods to estimate pathogen accumulation [64] found this relation for viruses causing Rice Tungro Disease, so therefore may be useful to characterize the dynamics of *S*. *kunkelii* accumulation in maize hybrids differing in resistance to Corn Stunt.

This work identified resistance mechanisms to the vector *D*. *maidis* and to the pathogen *S*. *kunkelii* as components of Corn Stunt pathosystem. Hybrids differed in their level of resistance and target organisms of such resistance. DK72-10 showed antixenosis and antibiosis to *D*. *maidis*, DK79-10 was resistant to *S*. *kunkelii* and DKB390 expressed antixenosis to *D*. *maidis* and resistance to *S*. *kunkelii*. More than one mechanism and target organism (vector or pathogen) of resistance were identified with this strategy, which therefore provides the potential to combine them to obtain even more resistant hybrids. This is an advantage of this type of testing over natural infestations, where the observed response (incidence or severity of symptomatic plants across hybrids) does not allow to differentiate between these foregoing factors. Additionally, since *D*. *maidis* is also vector of MBSP [20] and MRFV [21], the resistance mechanisms to *D*. *maidis* would also reduce the damage caused by these other pathogens. In summary, the presence of resistant hybrids combined with the use of an effective method for effective differentiation may facilitate the selection of hybrids capable of reducing the negative impact of Corn Stunt.

## Supporting information

S1 File (XLSX)
